# An Interventional Study to Evaluate the Effect of Communication Skill Training and Its Perception Among Second-Year Undergraduate Medical Students

**DOI:** 10.7759/cureus.95819

**Published:** 2025-10-31

**Authors:** Tejas A Acharya, Sunita B Chhaiya, Gaurav G Kakasaniya, Yagnik A Vaghasia, Riya J Master, Uday Shankar Singh

**Affiliations:** 1 Department of Pharmacology, C. U. Shah Medical College and Hospital, Surendranagar, IND; 2 Department of Pharmacology, Dr. M. K. Shah Medical College and Research Center, Ahmedabad, IND; 3 Department of Community Medicine, Pramukhswami Medical College, Karamsad, IND

**Keywords:** aetcom, communication skill training, kalamazoo checklist, simulated patients, undergraduate medical students

## Abstract

Background

Communication is a core competency in medical education, directly influencing patient trust, diagnosis, and adherence. Structured training interventions are recommended, but evidence from undergraduate cohorts in India remains limited. The objective of this study is to evaluate the effectiveness of structured communication skill training and explore students’ perceptions regarding its importance in medical education.

Materials and Methods

A prospective educational interventional study was conducted among second-year undergraduate students at a tertiary care teaching hospital to evaluate the impact of structured communication skill training. Communication performance was assessed using the Kalamazoo Essential Elements Communication Checklist (KEECC) before and after training, in which all seven components were intact but sub-questions were reduced for objectivity. A post hoc expert review and content validity index confirmed tool validity. Students’ perceptions toward communication skills were collected through an adapted questionnaire and analyzed for reliability and construct validity. The intervention consisted of scripted role plays demonstrating poor and effective communication. Data were analyzed using the Wilcoxon signed-rank and paired t-tests, with reliability (Cronbach’s alpha), factor analysis, and effect sizes (Cohen’s d and w) calculated.

Results

Of 92 eligible students, 79 (85.9%) completed the study. The mean KEECC score improved significantly from 3.04 to 7.36 out of 10 post-training (Z=-7.728, p<0.0001). All seven communication domains showed significant gains (p<0.001 each), with large effect sizes for building relationships (d=1.54), gathering information (d=1.36), reaching agreement (d=1.18), and providing closure (d=1.04). Perception analysis revealed a strong student endorsement: 75 (93.8%) agreed that communication skills build patient confidence, and 80 (100%) found the training effective. Factor analysis confirmed a unidimensional construct reflecting the perceived importance of communication skills.

Conclusion

Structured communication skill training significantly enhanced undergraduate medical students’ communication performance at our institute, with robust improvements across all domains. Moreover, students strongly recognized the importance and effectiveness of communication skill training.

## Introduction

Good communication skills are one of the required qualities for all doctors, including first-contact physicians [1]. Good communication skills are helpful in securing patients’ complaints and history, which in turn help deliver appropriate treatment [2]. Furthermore, it is the best way to establish a good doctor-patient relationship, which is very important in today’s unstable times. One of the best ways to implement this skill in students is by teaching it during undergraduate studies. In Indian medical education, the recent implementation of Competency-Based Medical Education (CBME) has introduced certain new modalities in the medical education curriculum. One of these is communication skills under the AETCOM (Attitude, Ethics and Communication) module among second-year undergraduate medical students [3]. Communication skill training can be provided to students by various methods, like demonstrating videos or performing role-play. A practical approach to communication can be provided by a patient-simulated method in which students are simulated as patients and communication with them can be assessed by various methods [4]. Few studies have evaluated communication skills training among undergraduate medical students. Reported approaches include assessing students’ attitudes using standardized scales and evaluating communication skills through simulated patient interactions [1,2]. However, comprehensive evaluations of structured training interventions remain scarce among Indian medical students.

This approach can serve as guidance for future doctors, and evaluating it can provide insight into communication skills for medical educators and policymakers. So this study was conducted with an overall aim of evaluating the effect of communication skill training in second-year undergraduate medical students in a tertiary care teaching hospital. The objectives of this study are to evaluate the effect of communication skill training and determine the perception of communication skill training amongst second-year undergraduate medical students.

This work is a part of Advance Course in Medical Education (ACME) and was presented as a poster at the second contact session of Advance Course in Medical Education (ACME) on July 12, 2025 at Pramukhswami Medical College, Karamsad.

## Materials and methods

Ethical consideration

The study was carried out at a tertiary care teaching hospital after obtaining written permission from the Institutional Ethics Committee (Human Research) of C. U. Shah Medical College, Surendranagar (approval No. CUSMC/IEC(HR)/Pub-30/2025/Final Approval/Out-57/2025).

Study design and setting

It was a prospective, educational interventional study carried out with second-year undergraduate medical students as participants.

Study population and place of study

Second-year undergraduate medical students in the Department of Pharmacology of a tertiary care teaching hospital.

Inclusion

All second-year undergraduate medical students who were willing to give written informed consent.

Exclusion

Students who remained absent from either the pre-training assessment, the post-training assessment, or the communication skill training sessions. However, all present students were allowed to attend each session even if they were excluded as per the stated criteria.

Sample size determination

As the study targeted the entire student population of the second-year undergraduate medical students, no prior sample size calculation was done. The participation rate justified the adequacy of the collected data.

Study instruments

Communication skill was assessed using the Kalamazoo Essential Elements Communication Checklist (KEECC), a validated and widely used tool for evaluating physician-patient communication [5]. It includes seven elements: building a relationship, understanding the patient’s perspective, opening the discussion, gathering information, sharing information, reaching an agreement, and providing closure. In the present study, all seven core elements of the KEECC were retained, while sub-questions were reduced and reworded for objectivity and feasibility. Each sub-question was rated as one (“done well”), 0.5 (“needs improvement”), or 0 (“not done”). A post hoc expert review was carried out for validity. A content validity index was calculated both at the item level (Item-level Content Validity Index (I-CVI)) and scale level (Scale-level Content Validity Index (S-CVI)) [6].

Students’ perceptions of communication skills and training were assessed with a questionnaire adapted from two prior studies [1,7]. One provided a validated scale, while the other contributed expert-reviewed items. The faculty members ensured clarity, and since this formed a new tool, post-study validation was conducted through exploratory factor analysis and Cronbach’s alpha.

Participant recruitment and data collection

Recruitment occurred during routine pharmacology practical sessions. Students were divided into batches and seated in separate rooms, with one investigator assigned per batch. The study was explained, written consent obtained, and a pre-training assessment conducted the same day to capture untrained skills. A patient-simulated approach was used, with students alternating in roles as a doctor and a patient. Each was first assessed as a doctor to minimize bias. Clinical scenarios were prepared, and patients drew lots describing symptoms. The set of clinical scenarios was the same for all the batches to ensure standardization. The investigator observed and rated communication using the KEECC.

Intervention

A structured training session was conducted in the lecture hall by investigators with departmental residents. Teaching was through scripted role-plays: one showing poor communication and another demonstrating effective communication. Student queries were addressed at the end of the session.

Post-Training Assessment

Post-training assessment followed the same procedure as the pre-training session, with the same batch and investigator allotments. Perceptions of communication skills and training were also collected through a Google Form (Google LLC, Mountain View, CA, USA), shared with all present students regardless of participation in the assessments.

Statistical analysis

Data were compiled in Microsoft Excel (Microsoft Corp., Redmond, WA, USA) and analyzed descriptively and inferentially. Categorical variables were expressed as frequencies and percentages. Pre- and post-training KEECC scores were compared using the Wilcoxon signed-rank test, with p<0.05 considered significant. Component scores were also compared using paired t-tests, and effect sizes were estimated with Cohen’s d (0.2 small, 0.5 medium, 0.8 large).

Perception data were analyzed for reliability and validity. Cronbach’s alpha assessed internal consistency (≥0.70 acceptable). Construct validity was examined through exploratory factor analysis using principal component analysis (PCA). Suitability was confirmed by the Kaiser-Meyer-Olkin (KMO) measure and Bartlett’s test. Factors with eigenvalues >1 and loadings ≥0.40 were retained; as a single-factor solution was expected, no rotation was applied. Chi-square goodness-of-fit tests were conducted to compare observed frequencies for each question (p<0.05 significant) and effect sizes were estimated by Cohen’s w (0.1 small, 0.3 medium, 0.5 large). All the analyses were performed in Microsoft Excel 2016, with confirmatory tests run in IBM SPSS Statistics for Windows, Version 24.0 (IBM Corp., Armonk, NY, released 2016).

## Results

**Figure 1:**
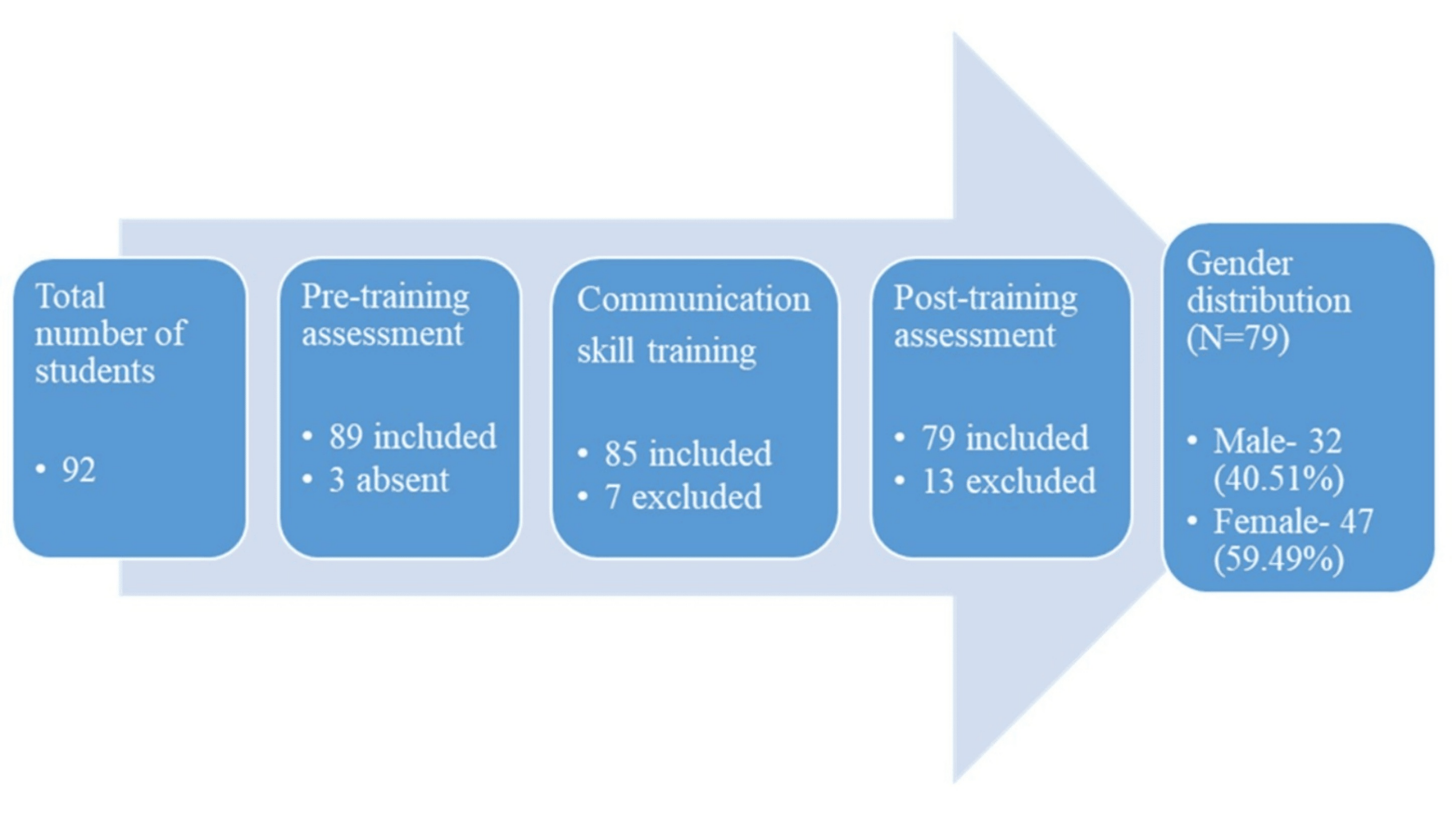
Flow of the participants through the study and gender distribution Data mentioned in the figure represent the number of students (N) and the percentage (%).

Out of 92 students of the second year, 79 completed the study. The achieved participation (N=92, 85.9%) provides a high participation rate, ensuring adequate representativeness of the study population. Out of 79 students who completed the study, 47 (59.49%) were women and 32 (40.51%) were men. The flow of participants through the study is depicted in Figure 1.

KEECC score analysis

**Figure 2:**
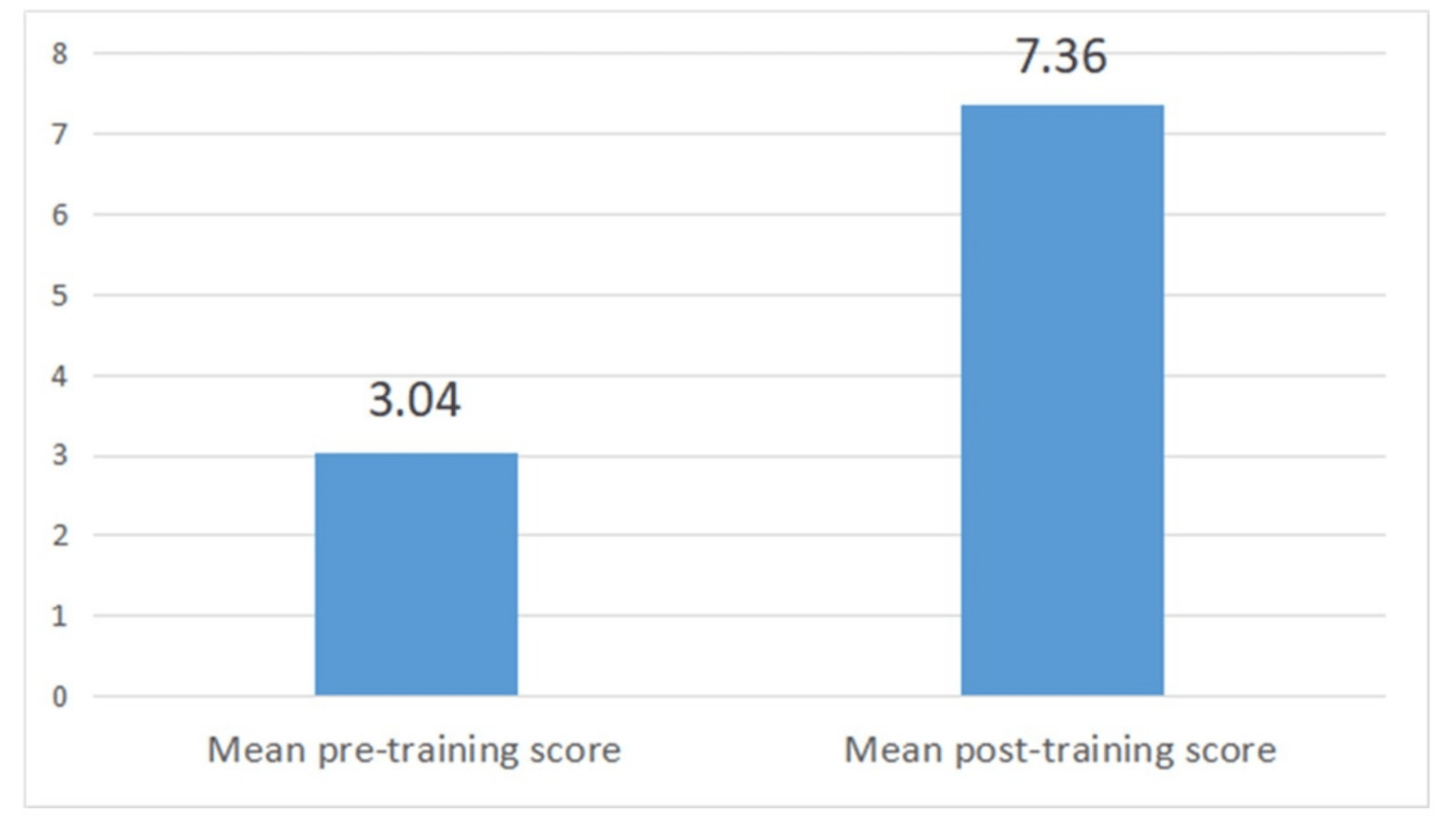
Mean pre- and post-training KEECC score KEECC : Kalamazoo Essential Elements Communication Checklist Data mentioned in figure indicate mean pre- and post-training KEECC scores out of 10.

The mean pre-training KEECC score was 3.04 out of 10, which was obtained by students before giving any exposure to communication skill training. The score was below 50%, suggesting students were struggling with simulated patients. The students achieved a mean post-training KECC score of 7.36 out of 10, which indicates good improvement after receiving communication skill training. The mean pre-training and post-training KEECC scores are illustrated in Figure 2.

On applying the Wilcoxon signed-rank test, this difference was found to be highly significant. (Z=-7.728, p<0.0001). This indicates communication skill training given to the students was highly effective in improving the KEECC score.

Further analysis of KEECC was done by analysis of each component of KEECC by paired t-test and effect size by Cohen’s d. The analysis of individual KEECC components revealed significant improvement across all domains following the training intervention (p<0.001 for each). Large effect sizes were observed for “Build a relationship” (d=1.54), “Gather information” (d=1.36), “Reach agreement” (d=1.18), and “Provide closure” (d=1.04), indicating substantial gains in these areas. The I-CVI for all the items was one, and S-CVI/AVE (Scale-level Content Validity Index/Average Proportion) was also one, indicating good relevance and excellent content validity. KEECC component analysis is outlined in Table 1.

**Table 1:** Pre and post-training analysis of KEECC components Data mentioned in table represent component wise mean pre and post-training score. *t value denotes value of paired t-test; **p<0.05 is kept as a statistically significant level; ***Cohen's d indicates standardized mean difference (effect size).

Question component	Total item score	Mean pre-training score	Mean post-training score	t value*	p- value**	Cohen’s d***
Build a relationship	1	0.42	0.96	-13.79	<0.001	1.54
Understand the patient’s perspective	1	0.86	0.99	-5	<0.001	0.55
Opens up the discussion	1	0.59	0.92	-6.92	<0.001	0.78
Gather information	1	0.11	0.72	-11.92	<0.001	1.36
Share information	3	0.56	1.66	-10.78	<0.001	0.78
Reach agreement	1	0.24	0.8	-10.45	<0.001	1.18
Provide closure	2	0.29	1.31	-12.77	<0.001	1.04

Perception questionnaire analysis

A perception questionnaire was filled out by 80 students regarding their perception of communication skill training. The questionnaire contained eight questions regarding overall attitude and requirements towards communication skill training.

Reliability Analysis

The internal consistency of the questionnaire was found to be good, with a Cronbach’s alpha value of 0.829, indicating that the items reliably measured the underlying construct.

Exploratory Factor Analysis

Exploratory factor analysis (EFA) was performed using principal component analysis, which is summarized in Table 2.

**Table 2:** Exploratory factor analysis of perception questionnaire KMO: Kaiser-Meyer-Olkin Measure of sampling adequacy; UG: Undergraduate Eigenvalues, percentage of variance, and cumulative percentage are reported for extracted components. Factor loadings represent correlations of items with the extracted factor. *χ² value denotes value of chi-square test with (degree of freedom), ** p<0.05 is kept as a statistically significant level.

Test / Component / Item	Value / Loading	% of Variance	Cumulative %
KMO measure	0.790	–	–
Bartlett’s test	χ²(28)*=206.87, p<0.001**	–	–
Eigenvalues			
Component 1	3.733	46.66	46.66
Component 2	0.949	11.86	58.52
Component 3	0.796	9.95	68.47
Factor loadings			
Communication skill is an asset for gaining the confidence of patients.	0.599	–	–
Communication skill is useful for my future practice.	0.725	–	–
Communication skill is an excellent method for reaching a diagnosis.	0.664	–	–
Communication skill should be taught throughout the UG course.	0.629	–	–
Communication skill can avoid conflicts and medico-legal issues.	0.601	–	–
Learning communication skills improves communication with patients.	0.774	–	–
Learning communication skill is as important as knowledge of medicine.	0.689	–	–
Communication skill training provided to us was effective.	0.759	–	–

The Kaiser-Meyer-Olkin (KMO) measure of sampling adequacy was 0.79, indicating that the sample was adequate for factor analysis. Bartlett’s test of sphericity was significant (χ²=206.87, p<0.001), confirming that correlations among items were sufficient to proceed with EFA. The eigenvalue criterion indicated that only one factor had an eigenvalue greater than one (3.73), which accounted for 46.7% of the total variance. Component matrix evaluation revealed that all eight items loaded substantially on this single component (loadings ranging from 0.60 to 0.77), suggesting a unidimensional structure representing students’ perceived importance of communication skills. The highest loading was observed for “Learning communication skills improves communication with patients” (0.774), followed by “Communication skill training provided to us was effective” (0.759).

Perception Questionnaire Response and Association Analysis

The response to perception questions was measured on a Likert scale ranging from "strongly disagree" to "strongly agree." The response to each question and its analysis by chi-square test are provided in Table 3.

**Table 3:** Perception questionnaire analysis df: degree of freedom; UG: undergraduate. Data mentioned in the table represent the number of students (N) and the percentage (%). *χ²  value denotes value of chi-square test,** p<0.05 is kept at a statistically significant level, ***Cohen's w indicates standard effect size of chi-square test.

No.	Question	Strongly disagree	Disagree	Neutral	Agree	Strongly agree	χ² (df) value*	p-value**	Cohen’s w***
1	Communication skill is an asset for gaining the confidence of patients	0 (0%)	1 (1.3%)	4 (5%)	36 (45%)	39 (48.8%)	97.125 (4)	P< 0.0001	1.10
2	Communication skills are useful for my future practice	0 (0%)	0 (0%)	3 (3.8%)	23 (28.7%)	54 (67.5%)	135.87 (4)	P<0.0001	1.30
3	Communication skill is an excellent method for reaching a diagnosis	0 (0%)	0 (0%)	1 (1.3%)	38 (47.5%)	41 (51.2%)	115.375(4)	P<0.0001	1.20
4	Communication skills should be taught throughout the UG course	0 (0%)	0 (0%)	3 (3.8%)	45 (56.3%)	32 (40%)	111.125 (4)	P<0.0001	1.18
5	Communication skills can avoid conflicts and medico-legal issues	0 (0%)	0 (0%)	10 (12.5%)	44 (55%)	26 (32.5%)	89.5 (4)	P<0.0001	1.06
6	Learning communication skills will improve my ability to communicate with patients effectively	0 (0%)	0 (0%)	0 (0%)	38 (47.5%)	42 (52.5%)	120.5 (4)	P<0.0001	1.23
7	Learning communication skills is as important as developing knowledge of medicine	0 (0%)	1 (1.3%)	2 (2.5%)	44 (55%)	33 (41.3%)	109.375 (4)	P<0.0001	1.17
8	The communication skill training provided to us was effective.	0 (0%)	0 (0%)	0 (0%)	41 (51.2%)	39 (48.8%)	120.125 (4)	P<0.0001	1.23

It is evident from the table that a clear majority of students agreed or strongly agreed with all positively framed statements. For example, 75 (93.8%) agreed or strongly agreed that communication skills are an asset for gaining patients’ confidence, and 77 (96.2%) agreed that communication skills are useful for their future practice. The training itself was also perceived as effective, with 80 (100%) students agreeing or strongly agreeing. Chi-square goodness-of-fit tests were conducted for each item to examine whether the distribution of responses significantly deviated from a uniform pattern. The results indicated that all items showed statistically significant differences in response distribution (p<0.0001 for all). Effect size (Cohen’s w) for the analysis ranged from 1.06 to 1.30, all of which indicate very large effects according to Cohen’s benchmarks.

## Discussion

The findings of the present study highlight the effectiveness of structured communication skill training in improving communication competencies among second-year undergraduate medical students. The significant increase in post-test scores using the KEECC underscores the positive impact of the intervention. Component-wise evaluation further revealed that all domains of communication improved significantly, with particularly large effect sizes in building relationships, gathering information, reaching agreement, and providing closure. These findings suggest that targeted training not only improves overall communication skills but also strengthens specific competencies that are crucial for effective patient care. These results are consistent with previous studies that have emphasized the importance of formal training in communication as part of the undergraduate medical curriculum [1,2].

Communication is recognized as a cornerstone of effective clinical practice. It not only facilitates accurate history-taking and diagnosis but also enhances patient satisfaction and treatment adherence [5]. The implementation of the AETCOM module under the Competency-Based Medical Education (CBME) framework by the National Medical Commission (NMC) in India represents a progressive step toward addressing this critical aspect of medical education [8]. In line with this, our study supports the inclusion of early, structured communication training using interactive methods such as role-play and simulated scenarios.

The highly significant improvement in communication skills post-intervention (Z=-7.728, p<0.0001) suggests that even brief training sessions can yield measurable benefits. This aligns with the findings in other studies in which significant improvement in post-test score after communication skill training was observed [2,9,10]. The use of simulation and role-play in our intervention allowed students to actively participate, observe, and reflect, thus promoting experiential learning, a well-established pedagogical approach in medical education [11].

In addition to performance-based outcomes, the study also examined students’ perceptions of communication skill training. The perception questionnaire demonstrated good reliability (Cronbach’s α=0.829), and exploratory factor analysis confirmed its unidimensional structure, with all items loading strongly (>0.59) on a single factor representing the perceived importance of communication skills. This provides evidence of the validity of the instrument for assessing student attitudes. A majority of students recognized the relevance of communication skills for building patient trust (75, 93.8%), aiding diagnosis (79, 98.7%), and preventing medico-legal issues (70, 87.5%). Additionally, 77 (96.3%) students agreed that communication should be an ongoing focus throughout the undergraduate course. This resonates with other studies done by Tanwani et al. [12] and Jagzape et al. [13], indicating that repeated and reinforced exposure to communication training across clinical years is a requirement at the current time. The chi-square analysis confirmed that these positive perceptions were statistically significant across all items.

In our study, all students agreed that learning communication skills would enhance their ability to interact effectively with patients. This aligns with the University of Toronto TCom Program evaluation [14], where 98% of early-year medical students reported benefiting from communication skills training, with 78% rating the benefit as substantial. Similarly, Taveira‑Gomes et al. found that students valued and retained key communication skills like empathy and information gathering after structured clerkships [15].

Together, these results highlight both the objective improvement in student competencies and the strong learner receptivity toward structured communication skill training. The convergence of performance gains and positive perceptions suggests that such interventions are both impactful and well-accepted, supporting their integration early in the medical curriculum.

Limitations and future directions

The study was limited to a single institute, which may affect generalizability. Furthermore, long-term retention of communication skills was not assessed, and training outcomes were measured in simulated rather than real clinical encounters. Future research should involve multi-institutional cohorts, longitudinal follow-up to assess skill retention, and incorporation of patient feedback to provide a more comprehensive evaluation of communication competence.

## Conclusions

Structured communication skill training using role play and simulated patient interactions significantly improved the communication performance of second-year undergraduate medical students in our institute, with large effects observed across multiple domains of the KEECC. The perception questionnaire further confirmed these students’ strong recognition of the importance and effectiveness of communication training, supported by robust reliability and validity evidence. These findings reaffirm that communication skills are teachable, and structured interventions such as the AETCOM module are essential for preparing empathetic and competent future healthcare professionals.

## References

[1] Nayak RK, Kadeangadi DM (2019). Effect of teaching communication skills to medical undergraduate students: an exploratory study. Indian J Community Fam Med.

[2] Chavda N, Solanki P, Dhanani JV, Shah A, Patel N, Bhadiyadra S (2020). Assessment of clinical communication skills of medical students through the simulated patient approach. Journal of Medical Education for Future Demands.

[3] (2025). Medical Council of India. Attitude Ethics and Communication (AETCOM): Competencies for the Indian Medical Graduate. Indian Medical Graduate.

[4] Rider EA, Hinrichs MM, Lown BA (2006). A model for communication skills assessment across the undergraduate curriculum. Med Teach.

[5] Makoul G (2025). Essential elements of communication in medical encounters: the Kalamazoo consensus statement. Acad Med.

[6] YaghmaIe F (2003). Content validity and its estimation. Journal of Medical Education.

[7] Vijayasree M (2019). Perception of attitude, ethics and communication skills (AETCOM) module by first MBBS students as a learning tool in the foundation course. J Evid Based Med Healthc.

[8] (2025). National Medical Commission. Competency based undergraduate curriculum. Indian Medical Graduate. New Delhi: NMC.

[9] Nguyen SN, Pham HT, Vu LT, Pham TX, Gottlieb B (2023). Effectiveness of a new clinical and communication curriculum for medical students: result of a double-blinded randomized controlled educational trial. J Med Educ Curric Dev.

[10] Thakur P, Dhage S, Solanke P (2025). Interventional study on effectiveness of attitude, ethics and communication module in improving communication skills of undergraduate medical students in Maharashtra. International Journal of Community Medicine and Public Health.

[11] Nestel D, Tierney T (2007). Role-play for medical students learning about communication: guidelines for maximising benefits. BMC Med Educ.

[12] Tanwani R, Chandki R, Joshi A, Arora VK, Nyati P, Sutay S (2017). Perception and attitude of medical students towards communication skills lab and teaching module. J Clin Diagn Res.

[13] Jagzape TB, Jagzape AT, Vagha JD, Chalak A, Meshram RJ (2015). Perception of medical students about communication skills laboratory (CSL) in a rural medical college of Central India. J Clin Diagn Res.

[14] Shapiro SM, Lancee WJ, Richards-Bentley CM (2009). Evaluation of a communication skills program for first-year medical students at the University of Toronto. BMC Med Educ.

[15] Taveira-Gomes I, Mota-Cardoso R, Figueiredo-Braga M (2016). Communication skills in medical students - an exploratory study before and after clerkships. Porto Biomed J.

